# Acute care of spontaneous intracerebral hemorrhage

**DOI:** 10.1186/s42466-025-00454-4

**Published:** 2025-12-15

**Authors:** Vishank Arun Shah, Bhagyashri Bhende, Shubham Biyani, Rohan Mathur, Sung-Min Cho, Julian Bösel

**Affiliations:** 1 Division of Neurosciences Critical Care, Department of Neurology, Johns Hopkins University School of Medicine, Baltimore, USA; 2 Division of Neurosciences Critical Care, Anesthesiology and Critical Care Medicine, Johns Hopkins University School of Medicine, Baltimore, USA; 3 Department of Neurology, University Hospital Heidelberg, Heidelberg, Germany; 4 Department of Neurology, Friedrich-Ebert-Hospital, Neumünster, Germany

## Abstract

**Background:**

Acute spontaneous intracerebral hemorrhage (ICH) is a life-threatening neurological emergency that afflicts more than 3 million people worldwide each year and has the highest mortality and morbidity of all stroke types. Acute care of ICH patients is targeted towards reducing secondary brain injury by preventing hematoma expansion and alleviating elevated intracranial pressure (ICP) from hydrocephalus, midline shift, brain compression and perihematomal edema.

**Aim:**

To provide a practical standard operating procedure (SOP) for the initial evaluation and management of acute spontaneous ICH patients.

**Method:**

This SOP was developed using the latest clinical guidelines and relevant studies on the management of ICH patients along with the authors' own experience and judgment.

**Results:**

Emergent care of ICH patients begins with stabilizing vital functions, rapid systolic blood pressure lowering and simultaneous reversal of any coagulopathy. Code ICH is a novel proposal to incorporate time-based bundled care to ensure timely institution of these therapies within 60 min of presentation. Clinical signs of elevated ICP and herniation should warrant prompt hyperosmolar therapy and emergent ventricular drainage for hydrocephalus. Emergent craniotomy or decompressive craniectomy for mass effect can be a lifesaving measure but may not improve functional outcomes. Early minimally invasive surgical interventions to promote clearance of intraventricular and parenchymal hemorrhage hold promise in not only improving survival but also promoting long-term functional improvement. Most importantly, early therapeutic nihilism must be avoided, and prognostication should be delayed for the first few days to allow time for recovery.

**Conclusion:**

Avoiding early pessimism and promoting emergent aggressive bundled care for ICH patients can promote favorable outcomes. Minimally invasive surgical interventions to promote prompt blood clearance should be considered to improve long-term recovery.

## Introduction

Acute spontaneous intracerebral hemorrhage (ICH), defined as non-traumatic bleeding within the brain parenchyma, occurs globally at a rate of 24.6 per 100,000 individuals [1, 2]. Overwhelmingly, ICH is a consequence of small vessel vasculopathy—either involving deep perforators in hypertensive microangiopathy or cortical vessels in cerebral amyloid angiopathy [2]. ICH may also result from macrovascular causes such as ruptured saccular aneurysms, arteriovenous malformations, cavernomas, mycotic aneurysms or venous congestion from cerebral venous sinus thrombosis [2]. ICH may also occur in association with oral anticoagulant use, particularly among the elderly [2, 3]. Often, multiple etiologic factors co-exist in the same patient, requiring a multi-pronged approach to the diagnosis and management of acute spontaneous ICH patients.

ICH has the highest early mortality rate of all stroke types, amounting to 35–40% in the first month [3, 4]. This high mortality is largely driven by early care limitations related to a perceived poor prognosis, and typically associated with admission severity factors such as hematoma volume, intraventricular extension, hemorrhage location, age, and Glasgow Coma Scale (GCS) [4–7]. This early prognostic nihilism has been challenged by more recent data that indicates a gradual but steady functional recovery over more protracted time periods, even among severe ICH survivors with severe early disability [8].

### Standard operating procedure for acute care of spontaneous ICH patients

**Fig. 1:**
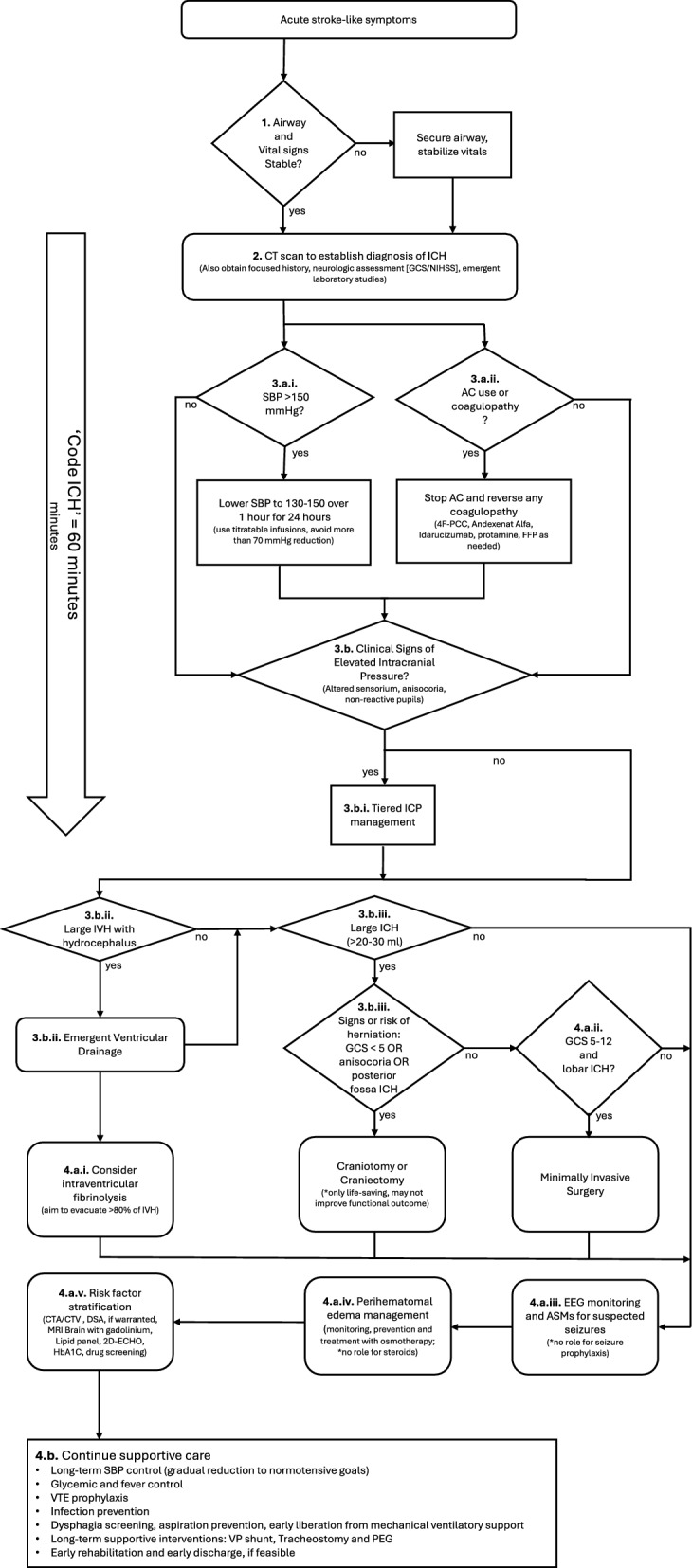
Standard Operating Procedure for the Acute Care of Intracerebral Hemorrhage (ICH). This figure shows a flow diagram demonstrating a standardized operating procedure for the acute care of ICH patients and corresponds with the description in the text of the manuscript. The care of ICH patients begins with stabilization of vital function, imaging confirmation of diagnosis, rapid blood pressure reduction, reversal of coagulopathy and if necessary, emergent bedside interventions to lower ICP, all of which should be pursued ideally within the first 60 min. This should be followed by neurosurgical evaluation for possible emergent neurosurgical interventions to assist with treating early neurological sequelae, such as raised intracranial pressure, hydrocephalus, mass effect and/or minimally invasive interventions to reduce hemorrhage volume. Subsequent care continues in the intensive care unit with continued blood pressure control, supportive care, prevention and management of medical complications. Etiologic workup and rehabilitation should begin after the hyperacute phase to ensure control of bleeding source and improving long-term recovery

The following standard operating procedure (SOP) is intended to provide an outline for the emergent and acute management of an adult patient with acute spontaneous ICH in the emergency room and the first days after admission based on current guideline recommendations [9–13]. The SOP description below corresponds to the flowchart outlined in Fig. 1. This SOP is not an established guideline or an expert consensus, and it includes an element of subjectivity informed by the clinical experience of the authors. Additional considerations, including rehabilitation, recovery, psychosocial disorders and outcome prognostication, are outside the scope of this article and will only be briefly discussed.

### Airway, breathing and circulation

Like all strokes, acute spontaneous ICH is a life-threatening neurological emergency. Akin to other emergent clinical scenarios, the acute care of any patient presenting with a suspected stroke must begin with an assessment of ongoing or impending cardio-respiratory compromise:

- Check and stabilize vital cardiac parameters including blood pressure and heart rate.
- Look for airway compromise, often related to altered sensorium in ICH patients, and secure airway and respiratory function when necessary.

### Establishing a diagnosis

- *History and physical examination*: The clinical presentation of acute ICH is most commonly characterized by acute focal neurological deficits that may differ from those caused by acute ischemic stroke somewhat in their constellation (e.g. less correlation with brain vessel territories) or their dynamics. The associated symptoms of ICH include headache, nausea, vomiting and altered consciousness [3]. A focused history should be taken to delineate the time since onset of neurological symptoms, presence of associated symptoms and screening for risk factors such as anticoagulant use, vascular co-morbidities and substance use [9]. A focused neurological examination using standardized scales such as the GCS and the National Institutes of Health Stroke Scale can help promptly gauge the severity of injury and provide quantifiable information for standardized communication. Unresponsive patients should also be screened for anisocoria or pupillary non-reactivity to identify ongoing or impending cerebral herniation. However, no clinical feature on history or examination can reliably distinguish ICH from ischemic stroke, and neuroimaging is necessary to establish the diagnosis [9].
- *Neuroimaging*: Emergent head computed tomography (CT) is the gold standard to diagnose ICH and should be pursued promptly [9]. The initial CT scan also provides important severity data such as the ICH volume, which is strongly associated with outcomes after ICH [5, 9, 14]. Such data may guide need for emergent surgical interventions such as ventricular drainage for intraventricular hemorrhage (IVH) and obstructive hydrocephalus or surgical decompression for midline shift/mass effect. Variable thresholds of ICH volume are associated with worse outcomes at different hematoma locations; however, in general, a hematoma volume of greater than 30 ml (ml) is consistently considered a poor prognostic marker [5, 14]. Therefore, for the purpose of this article, the authors define ICH severity as:
  Mild-to-moderate (or small volume) ICH: Hematoma volume <30 ml.
  Severe (or large) ICH: Hematoma volume >30 ml.
  Additional imaging to consider includes a CT angiogram (CTA) of the head, not only to detect macrovascular causes of ICH (see below), but also to assess for radiological markers of impending hematoma expansion, such as the ‘spot sign’, indicating active contrast extravasation within the hematoma [9, 15]. Presence of heterogenous densities within the hematoma on non-contrast CT, as indicated by the ‘blend’, ‘black hole’ and ‘swirl’ signs, may also suggest a heightened risk of hematoma expansion [14, 16].
- * Emergent laboratory testing*: Emergent screening for coagulopathy with prothrombin time, international normalized ratio (INR), partial thromboplastin time (PTT) and a platelet count is necessary for all patients with ICH to identify and mitigate the risk of hematoma expansion 9. Specific factor Xa inhibitor levels for newer oral anticoagulants are not routinely available for emergent testing to guide decision-making but can be considered in situations where historical confirmation of use of such medications is not feasible. Additionally, blood glucose levels, serum creatinine and troponin levels should be checked, given the association of derangements in these parameters with poor outcomes after ICH [9].

### Emergent treatment

Emergent treatment of ICH focuses on preventing early secondary brain injury and can be broadly divided into two categories: (1) preventing hematoma expansion and (2) managing mass effect and intracranial pressure (ICP) elevation.

#### Prevention of hematoma expansion

i. Blood pressure control: Acute hypertensive emergency (systolic blood pressure [SBP] > 180 mmHg) is extremely common in ICH patients and is strongly associated with hematoma expansion within the first 2–6 h from ictus, an important predictor of mortality and poor outcomes after ICH [2, 9]. Therefore, instinctively, acute SBP lowering is warranted in ICH, but clinical trials assessing this question have demonstrated conflicting results. The greatest benefit has been noted in patients with mild-to-moderate ICH and in the hyperacute phase, wherein the risk of hematoma expansion is the highest [2, 9, 12, 17]. Clinical trials investigating the safety and efficacy of SBP lowering after ICH have largely excluded patients with severe ICH (hematoma volume > 30 mL). Thus, there is limited evidence to support aggressive SBP reduction in this subgroup of severe ICH patients, wherein the intracranial pressure (ICP) may be elevated and SBP lowering may lower the cerebral perfusion pressure (CPP) and propagate secondary ischemic injury, and should be pursued with caution [9, 12]. With respect to timing, SBP lowering should be initiated and target SBP achieved within the first 6 h from symptom onset, and ideally within the first 2 h [9, 12]. In a pooled post-hoc analysis of the four INTERACT trials, the effect of SBP lowering on functional outcomes varied with time, with a significant improvement in outcomes when initiated within the first ~ 2 h and a trend towards an inverse effect on outcomes in participants randomized at later time points, highlighting the importance of early and rapid SBP lowering in these patients [18]. SBP variability within the first 24 h is associated with worse functional outcomes [19] and thus SBP reduction should be ideally achieved with gradual titration of continuous vasoactive infusions (e.g. nicardipine, clevedipine, urapidil, nitroprusside, labetalol or other rapidly titratable drugs), to avoid large fluctuations and significant variability in SBP [9, 12]. There is limited consensus on a specific SBP target in all ICH patients. As summarized above, in patients with mild-to-moderate ICH presenting within the first 6 hours, who are at a higher risk of hematoma expansion and a relatively lower risk of cerebral hypoperfusion from acute SBP lowering, SBP may be safely lowered to ≤140 mmHg [12] over the first hour after presentation to the emergency room, with the goal of maintaining the SBP within a range of 130-150 mmHg and strictly avoiding reductions below 110 mmHg to avoid secondary ischemic injury [9, 12]. Particularly in patients presenting with an SBP>220 mmHg, it is prudent to avoid more than a 70 mmHg reduction in SBP in the first hour, given its association with secondary ischemic injury [9, 12, 20]. There is limited evidence regarding the safety of this SBP threshold or any other specific threshold in patients with severe ICH or those needing surgical evacuation [9, 12, 20]. Recent evidence indicates a significant improvement in functional outcomes with pre-hospital lowering of SBP in ICH patients. However, the same trial showed harm for ischemic stroke patients and, thus, should not be pursued without confirming the diagnosis of ICH [21], highlighting the need for approaches to rapidly distinguish ICH from ischemic stroke patients in the pre-hospital setting, potentially with the help of mobile stroke units or biomarker-based detection of ICH [22], such as using point-of-care glial fibrillary acid protein [23], in the field.
ii. *Reversal of coagulopathy:* Anticoagulant-associated ICH accounts for nearly 20% of cases, with a three- to six-fold increase in risk of hematoma expansion and worse outcomes when compared to non-anticoagulant ICH [2, 3]. Patients with ICH secondary to newer direct oral anticoagulants, particularly factor Xa inhibitors, have better outcomes when compared to vitamin K antagonists [2, 3]. Nonetheless, prompt discontinuation of anticoagulant medications and rapid reversal of coagulopathy are core components of the emergent care of ICH patients [9, 12, 13] as follows:
  Vitamin K antagonists: Four factor prothrombin complex concentrate (4F-PCC) and intravenous vitamin K is preferred [9, 13].
  Factor Xa inhibitors: Andexenat alfa or 4F-PCC or activated PCC is preferred [9. In a patient presenting within 12 h of symptom onset and 15 h of the last dose of factor Xa inhibitor, if readily available, Andexenat alfa may be preferred to reduce hematoma expansion, although it may carry an increased risk of ischemic events [9, 12].
  Dabigatran: Idarucizumab; if not available, 4F-PCC or activated PCC is preferred [9, 13].
  Unfractionated heparin or enoxaparin: Protamine is the preferred agent [9, 13].ICH in the setting of other coagulopathies, such as chronic liver cirrhosis, disseminated intravascular coagulation (DIC), thrombolytic exposure or hemophilia are rare and beyond the scope of this SOP. However, any coagulopathy must be corrected with 4F-PCC, fresh frozen plasma, vitamin K (for cirrhosis and prolonged PTT/INR), cryoprecipitate (for DIC/thrombolytics/low fibrinogen levels) and/or specific coagulation factors (e.g. factor VIII for hemophilia A), with the goal to normalize and maintain coagulopathic laboratory parameters within normal ranges using serial laboratory monitoring.
iii. *Antiplatelet effect reversal*:The effect of antiplatelet agents on outcomes after ICH is much less clear [2]. Platelet transfusions to reverse antiplatelet effects may be associated with harm in non-surgical ICH patients and should be avoided unless a surgical intervention is planned [9, 12]. Thrombocytopenia should be corrected with platelet transfusions to maintain a level greater than 50–100,000 per microliter of blood. The role of desmopressin in reversing antiplatelet agent effects is not known [2, 9, 12], but can be considered in patients with renal failure and uremic platelet dysfunction.
iv. *Bundled care and ‘Code ICH’*: Care bundles with time metrics have significantly enhanced outcomes in ischemic stroke patients and a recent clinical trial randomizing acute ICH patients to a care bundle including emergent interventions to prevent hematoma expansion, including lowering SBP, anticoagulant reversal, glycemic and temperature control within 1 h of presentation, was associated with improvement in 6-month functional outcomes [24]. Based on this data, the 2025 ESO guidelines provide an expert consensus statement supporting care bundles in ICH, that include early blood pressure lowering (< 140 mmHg) in mild-to-moderate ICH patients, glycemic control (110–140 mg/dl in patients without diabetes and 140–180 mg/dl in patients with diabetes), correction of fever (temperature below 37.5 °C) and reversal of coagulopathy within 1 h of treatment initiation, along with early neurosurgical consultation to determine the need for surgical interventions (discussed below), early dysphagia screening and avoiding placement of do not resuscitate orders within the first 24 h [12]. ‘Code ICH’ is a proposal to introduce time-based bundled care for acute ICH patients with a door-to-needle target time of 60 min for emergency reversal of anticoagulation and parenteral SBP control [10, 11]

#### Management of elevated intracranial pressure (ICP) and mass effect

Emergent care of ICH also involves addressing acute elevations in ICP that may stem from obstructive hydrocephalus related to IVH or extrinsic hematomal compression of the third or fourth ventricle. Additionally, large volume hematomas may also cause significant midline shift and brain compression. The presence of these factors should be suspected in ICH patients who present with or develop altered consciousness or coma, with or without anisocoria or pupillary non-reactivity, and must be confirmed with neuroimaging.

i. *Tiered ICP management*: If the clinical examination and imaging are concerning for elevated ICP, a tiered approach to ICP management should be pursued, including elevation of head-of-bed, placing neck midline, sedation, osmotherapy with mannitol and/or hypertonic saline [2, 3, 9] and transient hyperventilation as measures to rapidly lower ICP until more definitive surgical interventions can be instated [25]. Although, Code ICH has not typically included the role of emergent ICP therapies, we recommend that emergent interventions to lower ICP, as discussed above, should be included within the first hour.
ii. *Obstructive hydrocephalus*: To lower mortality, emergent external ventricular drainage (EVD) to assist with diversion of cerebrospinal fluid (CSF) and lowering ICP should be performed in patients with IVH and/or associated obstructive hydrocephalus and depressed level of consciousness [9, 12].
iii. *Surgical management of ICH*: Life-saving surgical interventions may be indicated in severe ICH patients with large volume hematomas who either are at risk for or have evidence of mass effect on imaging and/or clinical signs of elevated ICP such as depressed level of consciousness and/or anisocoria. These should be considered as follows:
  Emergent open craniotomy for ICH evacuation and/or decompressive craniectomy may be considered as life-saving measures in supratentorial ICH patients with clinical evidence of mass effect and elevated ICP, but may not promote functional independence and, patients and families should be counselled accordingly [9].
  Based on the results of the recent SWITCH trial [26], early decompressive craniectomy without hematoma evacuation may also be considered for non-comatose adults (< 75 years of age) with altered sensorium (GCS 8–13), large-volume deep ICH (hematoma volume 30–100 ml) and severe neurological deficits (NIHSS 10–30), within 72 h of symptom onset, to reduce the risk of death and severe dependence (modified Rankin scale score 5–6) [12, 26].
  Cerebellar ICH patients with hematoma volume ≥ 15 ml or with evidence of neurological deterioration, brainstem compression or hydrocephalus should be strongly considered for emergent suboccipital craniectomy with hematoma evacuation with/without EVD placement to reduce mortality [9, 12].

### Intensive care unit (ICU) supportive care

Most ICH patients, particularly those with moderate-to-severe ICH, IVH, hydrocephalus or infratentorial hemorrhage, should be admitted to an ICU at a facility with neurosurgical capabilities [9]. Frequent neurological checks to rapidly detect neurological deterioration should be pursued [9]. Small volume ICH patients who do not require continuous vasoactive infusions for blood pressure control may be admitted to a specialized intermediate care stroke unit [9, 12].

Care in the acute phase involves prevention and management of delayed neurological sequelae, such as IVH, seizures and perihematomal edema, as well as acute medical complications such as hyperglycemia, fever, infections and venous thromboembolism—all of which are associated with delayed mortality and worse outcomes in ICH patients [2, 3].

#### ICU management of ICH-related neurological sequelae

i. * IVH treatment*: In addition to emergent EVD placement to divert CSF and lower ICP in patients with moderate-to-large IVH, promoting greater than 80% reduction in IVH volume improves survival and functional outcomes [2,8]. Intraventricular thrombolytic therapy with alteplase promotes faster clearance of IVH, improves survival and, when initiated within the first 48 hours of symptom onset, may also promote improvement in long-term functional outcomes, based on a recent individual patient data meta-analysis [2]. Therefore, after hematoma stability is established, early initiation of intraventricular alteplase should be strongly considered in patients with moderate-to-large IVH [9, 12], with a goal to promote >80% IVH removal. Combining intraventricular fibrinolysis with lumbar drainage of CSF has been shown to reduce shunt dependency in a phase-2 trial and may hold promise in ICH/IVH patients with persistent hydrocephalus, although additional research is warranted [27]. Minimally invasive endoscopic surgical evacuation of IVH may improve outcomes and reduce shunt dependency and can be considered [12].
ii. *Minimally invasive surgery (MIS)*: Minimally invasive endoscopic evacuation or stereotactic evacuation with or without targeted thrombolysis of the hematoma has been the subject of recent investigations showing significant mortality benefit, but continued uncertainty regarding functional outcome benefit in ICH patients [2, 9, 11]. The recently published ENRICH trial demonstrated improved 6-month functional outcomes with early MIS (within 24 h of onset) in lobar hemorrhages [28]. Thus, patients with large supratentorial ICH (> 20–30 mL) and altered sensorium (GCS score 5–12), without clinical evidence of brain herniation, should be considered for MIS interventions to reduce mortality in all types of ICH [9, 12], and to improve functional outcomes in patients with lobar hemorrhage, ideally within 24 h of symptom onset [12, 28]. Other MIS approaches and MIS for deep hemorrhages are currently being investigated and hold promise in changing the paradigm of acute ICH care.
iii. *Seizures*: Patients with ICH, particularly with a lobar location, are at high risk for clinical and subclinical seizures, however prophylactic antiseizure medications have not been shown to improve outcomes and are not necessary [2, 9, 12]. In ICH patients with altered sensorium of unclear etiology, continuous electroencephalographic monitoring should be considered. If seizures or epileptiform discharges are identified, antiseizure medications should be initiated promptly [2, 9, 12].
iv. *Perihematomal edema*: A delayed threat associated with ICH is the development of perihematomal edema (PHE), which may begin as early as 4–6 h after onset and peak by 3–7 days [2]. PHE, an inflammatory response to the hemorrhage, is an independent predictor of functional disability after ICH and is associated with higher hematoma volume, higher SBP, fever and hyperglycemia [2]. Thus, patients with severe (large) ICH must be closely monitored during the at-risk period with frequent neurological assessments and serial neuroimaging. Bolus dose hyperosmolar therapy remains the mainstay of treatment for PHE. This therapy should be not used prophylactically but rather in response to symptomatic effects of edema [2, 9]. Anti-inflammatory and immunomodulatory agents to reduce PHE are currently under investigation; however, steroids may be associated with harm after ICH and should be avoided [9, 12]. Given the close relationship of hematoma volume and PHE, therapies that promote hematoma clearance, such as MIS, hold promise in preventing the development of PHE.
v. *Risk factor stratification*: While supporting ICH patients during the acute phase, evaluation of the etiology should be pursued simultaneously to reduce the risk of ICH expansion and to identify interventions for secondary prevention. A CTA and/or CT venography should be strongly considered in patients < 70 years old, with lobar hemorrhages or in those with lobar or deep hemorrhages without a history of hypertension, to rule out macrovascular causes [9]. A digital subtraction angiography (DSA) should be pursued to identify a macrovascular cause if the CTA is concerning for a macrovascular cause [12] or if the CTA is unrevealing despite a high suspicion for a macrovascular source, such as in patients with atypical location or morphology of hematoma, known or high risk of infective endocarditis (e.g. intravenous drug abuse, bacteremia), among others. The latest ESO guidelines recommend using diagnostic algorithms, such as DIAGRAM, for targeted imaging to improve the yield of accurate prediction of the etiology of ICH, particularly to determine need for DSA [12]. If no macrovascular source is identified, an MRI brain with and without gadolinium contrast may be pursued, particularly in ICH patients with lobar hemorrhages, to rule out cerebral amyloid angiopathy, cavernous malformations, malignancy, among other causes 9. Additional workup includes toxicology screening, particularly in young ICH patients with no significant co-morbidities. Laboratory testing for vascular risk factors—such as lipid profile, hemoglobin A1C, echocardiography and telemetry monitoring—is warranted in all stroke patients, including ICH patients.

#### ICU management of medical complications and supportive care

i. *Hypertension*: There is limited data to support specific recommendations on SBP goals after the first 24 h. In general, SBP may be maintained below 160 mmHg with gradual up-titration of oral blood pressure medications after the acute period, with a long-term goal of normotension (< 130/80 mmHg) in the outpatient setting12.
ii. *Hyperglycemia and fever*: Hyperglycemia and fever may be associated with higher the risk of PHE, hematoma expansion and potentially worse outcomes 2. However, there is limited data to support aggressive correction of hyperglycemia and temperature control as individual therapeutic interventions for ICH patients12, but reasonable to control hyperglycemia (maintain blood sugar below < 180 mg/dl) while avoiding hypoglycemia, and fever (maintain temperature below 37.5 °C) as a part of a care bundle 9,12.
iii. *Venous thromboembolism*:* (VTE) prophylaxis*: Symptomatic VTE may occur in up to 5% of ICH patients, and routine screening yields a prevalence of 25% 2. Thus, VTE prophylaxis with intermittent pneumatic compression devices, graded compression stockings and/or elastic compression stockings should be initiated on the day of admission9,12. After ensuring hematoma stability, subcutaneous low-dose unfractionated heparin or low molecular weight heparin should be initiated within 24–48 h of ICH onset, particularly in immobile patients 2,9.
iv. *Dysphagia, airway compromise, mechanical ventilation and infections*: Dysphagia, sepsis, ventilator-associated pneumonia and prolonged mechanical ventilation duration are independent predictors of mortality as well as poor long-term recovery after ICH 8. Standard stroke and ICU care bundles for dysphagia screening, aspiration prevention, screening and prevention of sepsis and ventilator-associated pneumonia as well as early liberation from mechanical ventilation must be applied aggressively to critically ill ICH patients to improve likelihood of long-term recovery.
v. *Long-term supportive interventions*: Recovery of protective bulbar reflexes (swallowing and cough reflexes) after ICH may be protracted. Therefore, ICH survivors should be evaluated for tracheostomy and gastrostomy tube placement, particularly if there are no signs of recovery in the first 2 weeks after ICH. The SETPOINT-2 randomized controlled trial did not demonstrate any improvement in functional outcomes with early (~ 4 days) versus delayed tracheostomy (~ 11 days), with a lower need for tracheostomy in the delayed arm; these results suggest that it may be reasonable to wait for longer periods before committing a patient to a tracheostomy 29. Although need for gastrostomy is independently associated with poor long-term functional outcomes after ICH 8, the impact of such supportive interventions on the overall short- and long-term quality of life of ICH survivors is not clear. Approximately 20% of ICH patients with obstructive IVH may also need permanent CSF shunting 30. Longer duration of EVD, ICP spikes > 30 mmHg and greater daily CSF output are factors associated with need for permanent CSF shunting and should alert clinicians for neurosurgical referral 30.
vi. *Rehabilitation* While early mobilization within the first 24 h may be harmful, initiation of multidisciplinary rehabilitation 24–48 h after symptom onset should be considered for all ICH patients 9. Routine screening for anxiety, depression, pain and cognitive dysfunction at discharge and serially during outpatient follow-up should be considered, given the high prevalence of these disorders among ICH survivors.

### Prognostication: avoiding early therapeutic nihilism

Early care limitations in response to overly pessimistic prognostication using baseline ICH severity factors alone may lead to self-fulfilling prophecies among ICH patients, preventing patients from demonstrating the full scope of their recovery 2,3. The majority of commonly used ICH prognostication scales rely on admission characteristics alone and do not account for the patient’s clinical course and response to treatment and supportive care in the ICU. In a recent secondary analysis of two large clinical trials, more than 40% of severe ICH patients with severe disability at day 30 recovered to a good functional outcome by 1 year. The prediction of future recovery was significantly enhanced by incorporating hospital events and responses to therapy over baseline factors alone 2,8. Thus, it is imperative that early prognostication and withholding treatments based on such prognostication be avoided on the day of admission, particularly in the absence of prior directives from the patient 2,9,12. Prognostication and medical decision-making should be delayed for at least a few days after the insult. It is important to utilize a shared decision-making approach, incorporating the patient’s co-morbidities, ICH severity, hospital course, social support systems and personal preferences and not solely rely on commonly used outcome prediction scores based on admission characteristics only 2,9,12.

## Conclusions

Acute spontaneous ICH is a neurological emergency and warrants early aggressive interventions to prevent devastating complications, such as hematoma expansion, mass effect, elevated ICP, and herniation. Critical elements of emergent care of ICH include preventing hematoma expansion with rapid blood pressure control and reversal of coagulopathy, as well as treating elevated ICP with hyperosmolar therapy and emergent surgical interventions such as EVD and surgical evacuation. Code ICH is a proposal to standardize this emergent care with time-based metrics, ensuring that emergent interventions are delivered to patients within 60 min of arrival to the hospital. Minimally invasive surgery to promote rapid clearance of IVH and parenchymal hematomas show promise in improving long-term survival and functional outcomes after ICH. Avoiding early pessimism and pursuing early aggressive bundled care for all ICH patients can help improve outcomes after ICH.

## Data Availability

N/A.

## Abbreviations

CT: Computed Tomography
ICH: Intracerebral Hemorrhage
SBP: Systolic Blood Pressure
C: Anticoagulant
ICP: Intracranial Pressure
IVH: Intraventricular Hemorrhage
GCS: Glasgow Coma Scale
CTA: Computed Tomography Angiogram
CTV: Computed Tomography Venogram
DSA: Digital Subtraction Angiography
MRI: Magnetic Resonance Imaging
2D-ECHO2: Dimensional Echocardiogram
HbA1C: Hemoglobin A1C
EEG: Electroencephalogram
ASM: Anti-Seizure Medications
VTE: Venous Thrombo-Embolism
VP: Ventriculo-Peritoneal
PEG: Percutaneous Endoscopic Gastrostomy
